# Maximising Productivity and Eliminating *Campylobacter* in Broilers by Manipulating Stocking Density and Population Structure Using ‘Biosecurity Cubes’

**DOI:** 10.3390/pathogens10040492

**Published:** 2021-04-19

**Authors:** Genevieve Greene, Leonard Koolman, Paul Whyte, Helen Lynch, Aidan Coffey, Brigid Lucey, John Egan, Lisa O’Connor, Declan Bolton

**Affiliations:** 1 Teagasc Food Research Centre, Ashtown, 15 Dublin, Ireland; Genevieve.Greene@teagasc.ie (G.G.); leonard.koolman@teagasc.ie (L.K.); 2 School of Veterinary Medicine, University College Dublin, Belfield, 4 Dublin, Ireland; Paul.Whyte@UCD.ie (P.W.); Helen.Lynch@agriculture.gov.ie (H.L.); John.Egan@UCD.ie (J.E.); 3 Department of Agriculture, Food and the Marine, Backweston, Celbridge, W23 X3PH Kildare, Ireland; 4 Department of Biological Sciences, Munster Technological University, T12 T66T Cork, Ireland; aidan.coffey@cit.ie (A.C.); Brigid.Lucey@cit.ie (B.L.); 5 Food Safety Authority of Ireland, George’s Dock, 1 Dublin, Ireland; loconnor@fsai.ie

**Keywords:** *Campylobacter*, broilers, stocking density, productivity, biosecurity

## Abstract

This study investigates the effect of stocking density and population dynamics on broiler growth rates and productivity, while further validating the ability of the biosecurity cubes (BC) to protect birds from *Campylobacter*. In our methodology, six BC were constructed in a commercial broiler house containing approximately 28,500 birds. During three trials, the BC were stocked at densities of 12, 14, 16, 18, 20 and 22 birds/m^2^, with the main flock (20 birds/m^2^) considered the control. Periodically, 10 birds per density were weighed and examined. The *Campylobacter* status of the birds was monitored via faecal samples using the ISO 10272: 2017. The stocking density for maximum calculated yield was 20 (trials 1 and 2) or 22 birds/m^2^ (trial 3), followed by 18, 16, 14 and 12. At the stocking rate of 20 birds/m^2^, the birds in the pen grew faster than those at the same density in the main flock achieving 2 Kg 3–6 days faster. Birds in the BC were observed to be generally healthier, and in some cases, remained *Campylobacter* negative, even after the main flock was infected. Our results conclude that dividing the flock into sub-flocks of approximately 20 birds/m^2^ using BC could increase productivity up to 20%, while preventing *Campylobacter*.

## 1. Introduction

Stocking density and achieving the balance between productivity and bird welfare is an ongoing issue for the broiler industry. There are many publications exploring the effect of stocking density on broiler performance, carcass yield and meat quality, but direct comparison is complicated by differences in housing conditions, feed regimes, target bird weight, and even the way in which stocking density is expressed (birds or Kg of body weight (BW)/m^2^) [1]. The season may also have a significant effect, with lower optimal stocking densities in summer [2,3]. Some studies recommend lower stocking densities (fewer than 20 birds/m^2^), citing bird welfare issues [1,4,5], while others have reported negligible effects of similar or higher stocking densities on broiler health and welfare [6,7,8]. Thus, the scientific basis for recommending specific stocking densities are not unanimously agreed upon [9].

Regardless of stocking density, it is generally agreed that broilers perform better in smaller flocks. While the exact reasons for this are unclear, anecdotal evidence would suggest the group should be large enough to satisfy the anti-predator instinct to form protective groups, but small enough to allow for the establishment of a pecking order [10,11]. To the best of our knowledge, the effect of population dynamics (dividing the large commercial flock into smaller groups) on broiler performance has not been previously investigated.

Optimal broiler performance is also dependent on keeping pathogenic organisms away from the birds using effective biosecurity measures. Although considered to be primarily a human pathogen, *Campylobacter jejuni* may also infect broilers, causing reduced performance [12]. Moreover, campylobacteriosis is the most common cause of bacterial associated gastroenteritis in the European Union, where its incidence is conservatively estimated at nine million cases per annum with a disease burden of 0.35 million disability adjusted life years (DALY), costing €2.4 billion annually [13]. These bacteria preferentially colonise farmed poultry, and broilers are the primary source of human infections, accounting for 50–80% of cases [13,14]. A process hygiene criterion (PHC) for *Campylobacter* in broilers (Commission Regulation (EU) 2017/1495 of 23 August 2017 amending Regulation (EC) No 2073/2005) was, therefore, introduced in January 2018 in the European Union. Corrective actions are required if unsatisfactory results are obtained, including improvements in control activities on broiler farms. Thus, broiler producers require a biosecurity measure that will protect the birds against bacterial pathogens, especially *Campylobacter*, while also facilitating optimal broiler performance and welfare.

Our research group have previously developed an internal biosecurity structure to protect the broilers from *Campylobacter* [15]. This prototype consisted of four polycarbonate sheets (1 m high × 2.5 m long × 6 mm thick) supported at the corners by 4 × 1 m high wooden columns. Four slits (50 cm high × 8 cm wide), lined with industrial 50 mm thick bristle strips, allowed the feeder and drinker lines to run through the unit. The latest design, developed to facilitate better airflow over the birds and reduce the costs associated with upscaling, replaced the polycarbonate sheets with a galvanised steel mesh and a skirt of either polyurethane or fly-screen mesh. Although previously validated as an effective biosecurity measure to protect the birds against *Campylobacter*, additional validation research would provide further support for new investment in upscaled studies.

The primary objective of this study was to investigate the biosecurity cube (BC) as an effective measure to protect birds against *Campylobacter*, while also providing an opportunity to validate the effect the cubes have on stocking density and population dynamics on broiler growth rates and productivity. 

## 2. Results

The *Campylobacter* status of the broilers is shown in Table 1. In trial 1, the main flock (control) was *Campylobacter* positive after 35 days with faecal counts of 6.1 log_10_ cfu/g, in trial 2 the main flock was again positive after 35 days with faecal counts of 5.1 log_10_ cfu/g. In trials 1 and 2, *Campylobacter* was not detected in any of the samples obtained from within the biosecurity cubes. In contrast, in trial 3, all of the different broiler groups were infected with *Campylobacter* as early as 28 days. This was attributed to an issue with the temperature control system in the house where the fans which usually extract air from the house (thereby drawing air that enters the house through the vents at the side of the house, over the birds) were reversed, and large volumes of air were drawn directly into the house by these fans. 

In trial 1, the average time to achieve a mean broiler market weight of 2 Kg (the standard target weight for the broilers produced for the commercial poultry company that participated in our study) in the control (main) flock was approximately 32 d (Table 2). In the biosecurity cubes, the average time to achieve the same weight was approximately 28 days (12, 16, and 20 birds/m^2^), 27 days (14, and 18 birds/m^2^), and 32 days (22 birds/m^2^). These values (calculated on a per annum basis allowing for a 5 day turnaround between flocks, the shortest turnaround time possible) equate to reduced productivity of 32%, 19% and 5.5% at 12, 14 and 16 birds/m^2^ and increased yields of 5.5%, 20% and 10% at 18, 20 and 22 birds/m^2^, as compared to the control. A similar pattern was obtained in trial 2 as the time to 2 Kg was approximately 29, 31, 30, 30, 30 and 35 days at stocking densities of 12, 14, 16, 18, 20 and 22 birds/m^2^, respectively. As in trial 1, the highest annual productivity would be achieved with a stocking density of 20 birds per m^2^ (in the biosecurity cube). The predicted yield of 310,200 birds represents an increase of 14% as compared to the control. Biosecurity cube stocking densities of 18 and 22 birds/m^2^ also gave increased the predicted yields of 2% and 12%, respectively, while the lower stocking densities gave reduced bird harvests ranging from −9 to −28%. 

During trial 3, it took 30 days (12, 16, 20 and 22 birds/m^2^) and 29 days (14, and 18 birds/m^2^) to achieve 2 Kg in the biosecurity cubes. The predicted yields increased from 182,520 birds per annum at the lowest stocking rate (12) to 344,143 at the highest (22), and with the exception of 20 and 22 birds/m^2^ changing ranking places, the results were similar to trials 1 and 2.

Perhaps the most significant finding of these trials was the higher growth rates in the biosecurity cube, when compared to the main flock stocked at the same density (20 birds/m^2^), which resulted in a predicted 11% to 20% increase in productivity per annum. Birds in the biosecurity cube were observed to be generally healthier, and there was no difference in the mortality rates of the birds (data not shown) in the biosecurity cubes versus those in the main flock.

## 3. Discussion

Optimising stocking density for broiler production relies on achieving a broiler concentration that maximises productivity without compromising the health or welfare of birds. Higher stocking densities may result in increased moisture in the litter and higher ammonia emissions [11], which causes a higher prevalence and severity of footpad dermatitis and hock burn in birds [1]. Moreover, access to feeders and drinkers may be restricted, causing stress for the birds [11]. However, in our study, there was no visible evidence of distress, and the birds did not suffer from footpad lesions.

In this study, the optimal stocking density (in terms of overall productivity) in the biosecurity cubes was 20 birds/m^2^ (trials 1 and 3) and 22 birds/m^2^ (trial 3), and the target weight was achieved at least 3–6 days faster than the main flock, equating to a potential increase in productivity of approximately 30,000–60,000 birds per house per annum. These stocking densities are within the acceptable range of 15 to 23 birds/m^2^ reported by Dawkins et al. and 10 to 27 birds/m^2^ reported by Thaxton et al. [6,7]. The former study found no evidence of bird stress, as measured by corticosterone levels in faeces, at the higher stocking rates, while the latter authors reported that, in addition to stress hormones, blood glucose levels, plasma cholesterol concentrations, and nitrite levels were similarly unaffected by the higher stocking rates [6,7]. In modern broiler houses, the environment has more impact on welfare than stocking density [6]. Higher broiler densities can be achieved without adversely affecting bird health and welfare when the temperature and ventilation are continuously monitored and adjusted [16] and sufficient feeder space and nipple drinkers are provided [17]. Our study was undertaken in a modern broiler house with continuous temperature monitoring/adjustment, 36 to 65 birds per feeders and 7 to 12 birds per drinker. 

In the natural environment, birds instinctively form groups or flocks to reduce the risk of predation, allowing more time for foraging and rest which helps increase fitness and welfare [11]. In layer hens, Keeling et al. [18] reported that groups of fewer than 15 birds used aggression to establish dominance and maintain stable relationships, groups of 30 were too large for a stable hierarchy to develop, but too small for the tolerant social system that was observed in groups of 60 and 120 birds, where the birds were relatively non-aggressive [18]. Increased tolerance in larger groups (up to 200 birds) has also been observed in broilers [19]. Current broiler production systems in the EU are focused on maximising returns, and it is assumed that this is best achieved by housing thousands of birds in a single space. However, group size is an important and overlooked consideration. Our data clearly demonstrated (1) similar growth rates at 12, 14, 16, 18 and 20 birds/m^2^ (all three trials) and 22 birds/m^2^ (trial three only), where any differences in growth rates were at best marginal and (2) a faster growth rate in the biosecurity cubes even when the stocking density was similar to the main flock. Higher growth rates have been previously observed in smaller groups of broilers, but the magnitude of the differences depended on the study design, being more pronounced in smaller (50 to 200 birds) groups and/or at lower stocking densities (0.05 to 0.11 birds/m^2^) [19,20]. 

One possible explanation for the faster growth rate in the cubes, even when the stocking density was similar to the main flock, is improved access to feeders and drinkers [16,17], but the feeder and drinker to bird ratio was similar in the cubes stocked at 20 birds/m^2^ and those in the general flock. Other studies that observed higher growth rates in smaller broiler groups have also ruled out better access to resources [19,20]. Roosting behaviour, facilitated by simulated walls provided by the cube, could also account for improved performance, but this was only observed toward the last week of the study when the test birds were already bigger than the control flock [21]. Moreover, we have no evidence of less movement by the test birds. The explanation of this phenomenon is more likely related to the primary function of the biosecurity cubes. Broiler production is intensive, and the birds can suffer a range of infectious diseases. This and previous research have established that the biosecurity cubes provide protection against *Campylobacter* and presumably other diseases which are transmitted from bird to bird within the flock [15]. The improved growth performance may, therefore, at least in part, be due to controlling disease within the test broilers [12]. 

Considering food safety, the enhanced growth rate in the smaller sub-flocks would also reduce the slaughter age and associated risk of *Campylobacter*. Conventionally produced broilers are usually harvested when they achieve their target weight, which requires on average 41.4 days in the EU [13]. However, flock positivity is directly related to slaughter age, and the younger the birds, the less likely they are to be colonised by *Campylobacter* [22,23]. Van Wagenberg et al. estimated that if all flocks were slaughtered by 35 days or earlier, there would be a reduction in human campylobacteriosis by 10–18% [24]. Multivariate analysis of the EU baseline data found that the risk of colonisation increased approximately two-fold for every 10 day increase in the age of birds, and the overall incidence of campylobacteriosis in the human population would be reduced by 21% to 43% if the slaughter age was reduced to 28 days [25]. This is supported by Romero-Barrios et al., who estimated that there would be a 43% reduction in human cases if all broilers were slaughtered at age 27 days [26]. However, reducing the slaughter age to four weeks would only be implemented if birds reach the desired weight in that timeframe and there are no adverse health and welfare effects on the birds or financial loss for the farmer, as has been achieved in this study. Thus, using the biosecurity cubes to sub-divide the broiler population into smaller sub-flocks could increase productivity, while at the same time protecting the birds against *Campylobacter* through the provision of a physical barrier and reduced slaughter age. However, as illustrated in trial 3, the cubes will not protect against serious breaches in the overall biosecurity of the broiler house. It was concluded that dividing the flock into sub-flocks of approximately 250 birds using the biosecurity cubes and stocking at a rate of 20 birds/m^2^ could increase productivity by up to 20%, while helping to resolve the *Campylobacter* issue for the broiler sector. Future work will focus on up-scaling to further validate the productivity and protection effects observed in this study.

## 4. Materials and Methods

### 4.1. Description of the Farm Used in Study

This study was undertaken on a broiler farm in County Monaghan (Ireland) which had three broiler houses on site, as well as a separate housing facility for dairy cattle in the winter months. The broilers were species *Gallus gallus domesticus*, Ross breed. Two of the three broiler houses, as well as the cattle shed, were located adjacent to each other, while the third broiler house was situated on a separate concrete apron. It was this third broiler house that was chosen to be part of this study. The broiler house accommodated approximately 28,500 birds at a stocking density of approximately 20 birds/m^2^. Broilers contained within the shed were of mixed-sex. The bedding was medicated milled straw, chipped straw treated with antimicrobials, (Straw Chip Limited, Ballycullane, Athy, Kildare, Ireland) and spread to a depth of approximately 5 cm over the entirety of the production floor. The house used a fan-based system to control ventilation. Thinning or partial depopulation of the flock occurred around day 28, with final thin around day 35 or 36. Catching staff adhered to the industry standard biosecurity protocols (broiler suits, boot covers, hair nets etc.) and approximately half of the flock were removed. Birds within biosecurity cubes were not thinned until day 35 or 36. Trial 1 took place during December, trial 2 took place during March and trial 3 took place during August. 

### 4.2. Description of Biosecurity Cube Used in Study

**Figure 1:**
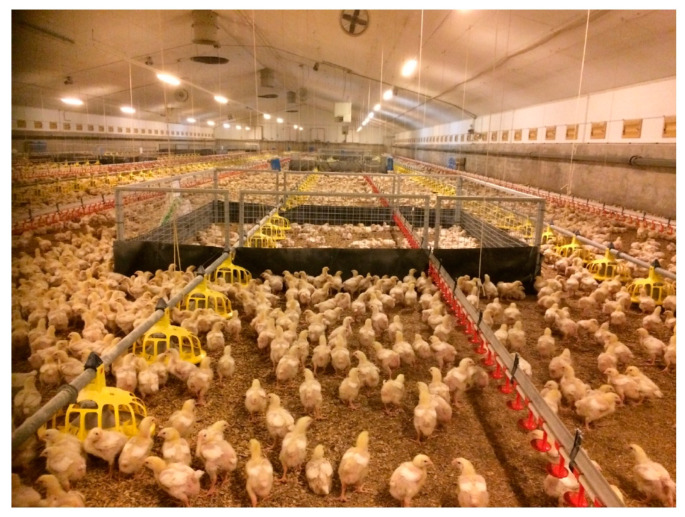
Biosecurity cube with polyurethane skirting lining the perimeter to limit the interaction between broilers inside and outside the cube.

The biosecurity cube, previously developed by our research team to protect broilers against *Campylobacter*, was used as the bird pen to segregate sub-populations of broilers within the main flock. Each cube was composed of four galvanised steel mesh panels (1 m high × 3.43 m long) (Cill Dara animal compounds limited, Kildare, Ireland) bolted at the corners (Figure 1). Slits in 2 ends accommodated the rise and fall of the feeder and drinker lines. This was encircled by either a polyurethane film (B&Q, DIY Store, Liffey Valley, Dublin, Ireland), or fly-screen mesh (200 cm high × 13.72 m) (Midge Mesh Roll, Goss Fly Screens, Louth, Ireland). The floor area within each cube was 11.76 m^2^, with four feeders and 21 nipple drinkers enclosed within this space. The six biosecurity cubes used in this study were assembled the day before stocking, when each cube was randomly stocked at a density of 12 (141 birds), 14 (165 birds), 16 (188 birds), 18 (212 birds), 20 (235 birds) or 22 (259 birds) birds/m^2^. Three cubes were placed evenly straddling the feeder and drinker lines of the right-hand side of the house, and three were placed straddling the feeder and drinker lines of the left-hand side of the house.

### 4.3. Monitoring the Bird Weights

Upon arrival (t = 0) and on days 4, 7, 11, 14, 18, 21, 25, 28, 32, and 35, 10 birds were randomly weighed in each biosecurity cube and in the main (control) flock using the BW-2050 weighing system (Weltech International Limited, Cambridgeshire, UK). The general health and wellbeing of the birds were assessed, and dead birds were removed and recorded. Boot covers and gloves were changed before entering each cube to prevent cross contamination between cubes. 

### 4.4. Sample Collection and Campylobacter Testing

Faecal samples were taken on days 0, 7, 14, 21, 28, and 33 or 35 days and tested for *Campylobacter* spp., including two composite (2 × 10 faecal samples) samples in each of the biosecurity cubes and five composite (5 × 10 faecal samples) samples in the remainder of the flock. Samples were transported to the laboratory and processed within 24 h. To detect *Campylobacter*, samples were both direct plated and enriched according to the Horizontal Method for Detection and Enumeration of *Campylobacter* spp. (ISO 10272: 2017) [27,28]. The composite faecal samples were tested by adding 10 g to 90 mL of Bolton enrichment broth (CM983B, Oxoid, Cambridge, UK) supplemented with 5% lysed horse blood (SR048C, Lennox, Dublin) and Bolton broth supplement (SR183E, Oxoid, Cambridge, UK), to give a 1:10 dilution and stomached for 60 s. After mixing, serial dilutions were prepared using maximum recovery diluent (MRD) (CM0733B Oxoid, Cambridge, UK), and 100 µL aliquots were plated out on modified CCDA (CM0739, Oxoid, Cambridge, UK) supplemented with CCDA selective supplement (SR0155, Oxoid, Cambridge, UK), and 1 mg/L of tazobactum sodium salt (Fisher Scientific, Dublin, Ireland) for each composite sample. Sample inoculated broths were also enriched at 42 °C for 48 h under microaerobic conditions using Anaero Jars (AG0025A, Fannin, Dublin) with Campygen atmosphere generation kits (CN025A, Oxoid, Cambridge, UK). Samples were plated out on tazobactum-supplemented mCCDA following incubation. All presumptive *Campylobacter* isolates were confirmed initially using the following tests; aerobic growth, L-alanine test (Oxoid Biochemical Identification System (O.B.I.S.), Thermo scientific, Hampshire, UK), oxidase test (Fisher Scientific, Dublin, Ireland), and growth on chromogenic agar (RAPID’ *Campylobacter* Medium, BioRad, Dublin, Ireland). After biochemical and chromogenic testing, a randomly selected representative cohort of isolates was speciated using a previously published conventional PCR method [29]. All isolates were stored at −70 °C in defibrinated horse blood (HB034, Cruinn Diagnostics, Dublin, Ireland). 

### 4.5. Data Analysis

The bird weight data was analysed by performing linear regression (plots of time v bird weights) using GraphPad Prism 7.02 (Graphpad Software Incorporated, San Diego, CA, USA). The entire study was repeated on three separate occasions, reported as trials 1, 2, and 3.

## Figures and Tables

**Table 1:** The *Campylobacter* status of the broilers in the main house and in the biosecurity cubes (BC).

Trial	1			2			3		
Stocking Density (Birds/m^2^)	^1^ Status	^2^ Time (d)	^3^ Count	Status	Time (d)	Count	Status	Time (d)	Count
20 (control)	+	35	6.1	+	35	NA	+	28	5.3
12	−	^4^ NA	NA	−	NA	NA	+	33	6.0
14	−	NA	NA	−	NA	NA	+	28	4.8
16	−	NA	NA	−	NA	NA	+	33	6.2
18	−	NA	NA	−	NA	NA	+	33	6.5
20	−	NA	NA	−	NA	NA	+	33	5.6
22	−	NA	NA	−	NA	NA	+	28	2.0

^1^*Campylobacter* status at the time of harvesting. ^2^ Time at which *Campylobacter* were first detected. ^3^ *Campylobacter* count (log_10_ cfu/g faeces) when first detected. ^4^ Not applicable.

**Table 2:** The effect of stocking density on the growth of broilers.

Stocking Density (Birds/m^2^)	20 (Control)	12	14	16	18	20	22
Trial 1							
^1^ Slope	0.062	0.072	0.06	0.071	0.067	0.075	0.064
SE	0.004	0.005	0.080	0.005	0.005	0.086	0.003
R^2^	0.96	0.96	0.97	0.96	0.96	0.96	0.98
^2^ Mean time (d) to 2 Kg	32.2	27.8	27.1	27.8	26.6	25.9	32
^3^ SD	0.34	0.18	0.20	0.13	0.14	0.11	0.18
^4^ Predicted yield	294,900	200,340	238,414	278,800	311,374	354,300	325,453
Predicted change (%)	-	−94,560 (−32%)	−56,486 (−19%)	−16,100 (−5.5%)	+16,474 (+5.5%)	+59,400 (+20%)	+30,553 (+10%)
Ranking	4	7	6	5	3	1	2
Trial 2							
Slope	0.057	0.070	0.064	0.066	0.066	0.066	0.058
SE	0.004	0.005	0.005	0.005	0.005	0.005	0.003
R^2^	0.95	0.95	0.95	0.96	0.95	0.95	0.97
Mean time (d) to 2 Kg	35.1	28.6	31.3	30.3	30.3	30.3	34.5
SD	0.36	0.17	0.14	0.26	0.24	0.27	0.17
Predicted yield	273,067	195,536	211,157	248,159	279,180	310,200	304,937
Predicted change (%)	-	−77,533 (−28%)	−61,910 (−23%)	−24,908 (−9%)	+6113 (+2%)	+37,133 (+14%)	+31,870 (+12%)
Ranking	4	7	6	5	3	1	2
Trial 3							
Slope	0.060	0.066	0.069	0.066	0.069	0.067	0.067
SE	0.004	0.005	0.005	0.005	0.006	0.004	0.005
R^2^	0.96	0.96	0.96	0.96	0.95	0.97	0.97
Mean time (d) to 2 Kg	33.3	30.3	29.0	30.3	29.0	29.9	29.9
SD	0.18	0.12	0.12	0.16	0.13	0.12	0.13
Predicted yield	280,800	182,520	225,540	243,333	289,853	312,857	344,143
Predicted change (%)	-	−98,280 (35%)	−55,260 (−20%)	−37,467 (−13%)	+9053 (+3%)	+32,057 (+11%)	+63,343 (+23%)
Ranking	4	7	6	5	3	2	1

^1^ Slope of the linear regression line of the plot of time v bird weights, prepared using GraphPad Prism 7.02 (Graphpad Software Incorporated, San Diego, CA, USA). ^2^ Mean time (in days (d)) to achieve 2 Kg. ^3^ SD = Standard deviation in bird mass at the sampling time closest to the target weight time. ^4^ Predicted yield per house (1500 m^2^) per annum.

## Data Availability

The data presented in this study are available on request from the corresponding author. The data are not publicly available due to privacy concerns from our commercial research partners.
